# Effect of dimethylamine on the gas phase sulfuric acid concentration measured by Chemical Ionization Mass Spectrometry

**DOI:** 10.1002/2015JD023868

**Published:** 2016-03-24

**Authors:** L. Rondo, S. Ehrhart, A. Kürten, A. Adamov, F. Bianchi, M. Breitenlechner, J. Duplissy, A. Franchin, J. Dommen, N. M. Donahue, E. M. Dunne, R. C. Flagan, J. Hakala, A. Hansel, H. Keskinen, J. Kim, T. Jokinen, K. Lehtipalo, M. Leiminger, A. Praplan, F. Riccobono, M. P. Rissanen, N. Sarnela, S. Schobesberger, M. Simon, M. Sipilä, J. N. Smith, A. Tomé, J. Tröstl, G. Tsagkogeorgas, P. Vaattovaara, P. M. Winkler, C. Williamson, D. Wimmer, U. Baltensperger, J. Kirkby, M. Kulmala, T. Petäjä, D. R. Worsnop, J. Curtius

**Affiliations:** ^1^ Institute for Atmospheric and Environmental SciencesGoethe University Frankfurt am MainFrankfurt am MainGermany; ^2^ CERNGenevaSwitzerland; ^3^ Department of PhysicsUniversity of HelsinkiHelsinkiFinland; ^4^ Laboratory of Atmospheric ChemistryPaul Scherrer InstituteVilligenSwitzerland; ^5^ Institute for Atmospheric and Climate ScienceETH ZurichZurichSwitzerland; ^6^ Institute for Ion Physics and Applied PhysicsUniversity of InnsbruckInnsbruckAustria; ^7^ Center for Atmospheric Particle StudiesCarnegie Mellon UniversityPittsburghPennsylvaniaUSA; ^8^ Kuopio UnitFinnish Meteorological InstituteKuopioFinland; ^9^ School of Earth and EnvironmentUniversity of LeedsLeedsUK; ^10^ Division of Chemistry and Chemical EngineeringCalifornia Institute of TechnologyPasadenaCaliforniaUSA; ^11^ Department of Applied PhysicsUniversity of Eastern FinlandKuopioFinland; ^12^ Department of Atmospheric SciencesUniversity of WashingtonSeattleWashingtonUSA; ^13^ Department of ChemistryUniversity of CaliforniaIrvineCaliforniaUSA; ^14^ CENTRA‐SIMUniversity of Lisbon and University of Beira InteriorLisbonPortugal; ^15^ Leibniz Institute for Tropospheric ResearchLeipzigGermany; ^16^ Department of Environmental ScienceUniversity of Eastern FinlandKuopioFinland; ^17^ Faculty of PhysicsUniversity of ViennaViennaAustria; ^18^ Aerodyne Research, Inc.BillericaMassachusettsUSA

**Keywords:** CLOUD experiment, nucleation, Chemical Ionization‐Atmospheric Pressure interface‐Time Of Flight Mass Spectrometer

## Abstract

Sulfuric acid is widely recognized as a very important substance driving atmospheric aerosol nucleation. Based on quantum chemical calculations it has been suggested that the quantitative detection of gas phase sulfuric acid (H_2_SO_4_) by use of Chemical Ionization Mass Spectrometry (CIMS) could be biased in the presence of gas phase amines such as dimethylamine (DMA). An experiment (CLOUD7 campaign) was set up at the CLOUD (Cosmics Leaving OUtdoor Droplets) chamber to investigate the quantitative detection of H_2_SO_4_ in the presence of dimethylamine by CIMS at atmospherically relevant concentrations. For the first time in the CLOUD experiment, the monomer sulfuric acid concentration was measured by a CIMS and by two CI‐APi‐TOF (Chemical Ionization‐Atmospheric Pressure interface‐Time Of Flight) mass spectrometers. In addition, neutral sulfuric acid clusters were measured with the CI‐APi‐TOFs. The CLOUD7 measurements show that in the presence of dimethylamine (<5 to 70 pptv) the sulfuric acid monomer measured by the CIMS represents only a fraction of the total H_2_SO_4_, contained in the monomer and the clusters that is available for particle growth. Although it was found that the addition of dimethylamine dramatically changes the H_2_SO_4_ cluster distribution compared to binary (H_2_SO_4_‐H_2_O) conditions, the CIMS detection efficiency does not seem to depend substantially on whether an individual H_2_SO_4_ monomer is clustered with a DMA molecule. The experimental observations are supported by numerical simulations based on A Self‐contained Atmospheric chemistry coDe coupled with a molecular process model (Sulfuric Acid Water NUCleation) operated in the kinetic limit.

## Key Points

- H_2_SO_4_ concentration measured by CIMS in CLOUD was influenced by the presence of dimethylamine
- CIMS's detection efficiency is not substantially influenced by the presence of dimethylamine
- Information of sulfuric acid cluster composition by CI‐APi‐TOF explains most of the observed effect

## 1 Introduction

Clouds play a key role in the Earth's climate. The climate forcing due to aerosol‐climate interaction causes the largest source of uncertainty in present climate models [*Intergovernmental Panel on Climate Change*, 2001]. Much of the uncertainty regarding the effect of clouds on climate arises from the complexity of cloud formation. One important source for the atmospheric particle population, which links directly to the cloud formation via the global cloud condensation nuclei [*Merikanto et al*., 2010], is nucleation [*Kulmala et al*., 2013]. The exact mechanisms for particle nucleation and growth are still under investigation since knowledge of the cluster composition is required to fully understand new particle formation.

One of the primary vapors responsible for nucleation is sulfuric acid (H_2_SO_4_) [*Kulmala et al*., 2004, 2006; *Riipinen et al*., 2007]. However, since the observed atmospheric particle formation in the boundary layer cannot be explained solely by the typical maximum daytime concentrations of sulfuric acid and water vapor [*Kirkby et al*., 2011], other compounds have to contribute to new particle formation. Recent ternary nucleation studies of the H_2_SO_4_‐H_2_O system along with ammonia [*Benson et al*., 2009; *Kirkby et al*., 2011], oxygenated organic compounds [*Zhang et al*., 2004; *Metzger et al*., 2010; *Riccobono et al*., 2012, 2014; *Schobesberger et al*., 2013], and amines [*Kurtén et al*., 2008; *Almeida et al*., 2013; *Kürten et al*., 2014] investigated the effect of these compounds on nucleation. Results from the CLOUD (Cosmics Leaving OUtdoor Droplets) experiment at CERN (European Organization for Nuclear Research) described nucleation enhancements of a factor up to ~1000 relative to the binary H_2_SO_4_‐H_2_O system, from the addition of atmospherically relevant concentrations of ammonia [*Kirkby et al*., 2011]. However, even considering the enhancement from ammonia and from ion‐induced nucleation, the achieved particle formation rates are still too small in comparison with most observations in the continental boundary layer [*Kirkby et al*., 2011].

In the continental boundary layer amines are present as trace gases with mixing ratios of less than 1 to a few tens of parts per trillion by volume [*Ge et al*., 2011a; *Hanson et al*., 2011], with occasional observations of ~100 pptv [*Freshour et al*., 2014] and have gained recent attention due to their potential contribution to nucleation or particle growth [*Kurtén et al*., 2008; *Berndt et al*., 2010; *Ge et al*., 2011b]. Recent experimental aerosol nucleation studies show that amines stabilize the nucleating clusters much more efficiently than ammonia [*Chen et al*., 2012; *Almeida et al*., 2013; *Kulmala et al*., 2013; *Jen et al*., 2014; *Kürten et al*., 2014]. According to *Almeida et al*. [2013], amines are able to play a key role in the very first steps of particle nucleation by enhancing the nucleation rate of sulfuric acid particles by more than a thousand times compared to the H_2_SO_4_‐H_2_O‐NH_3_ system. The capability of amines and sulfuric acid to form stable clusters in the neutral, i.e., uncharged, system at atmospherically relevant concentrations has recently been demonstrated by *Kürten et al*. [2014], who for the first time measured neutral clusters containing up to 14 sulfuric acid and 16 dimethylamine molecules during experiments at the CLOUD chamber.

In addition to laboratory experiments, theoretical studies using quantum chemical calculations have also focused on the role of amines in aerosol nucleation [*Kurtén et al*., 2008, 2011; *Ortega et al*., 2012; *Kupiainen‐Määttä et al*., 2013; *Loukonen et al*., 2014]. Among these theoretical studies, it has been suggested that the sensitivity of Chemical Ionization Mass Spectrometers (CIMS) toward gas phase sulfuric acid monomers (H_2_SO_4_) could be influenced by the presence of amines (e.g., dimethylamine (DMA), (CH_3_)_2_NH). In this way, the clustering between H_2_SO_4_ and (CH_3_)_2_NH leading to a H_2_SO_4_ **·**(CH_3_)_2_NH complex coupled to H_2_O molecules, abbreviated as SA**·**DMA in the following, could affect interpretation of the relationship between the derived sulfuric acid monomer concentration, the nucleation rate, and the growth rate [*Kurtén et al*., 2011; *Kupiainen‐Määttä et al*., 2013; *Loukonen et al*., 2014]. According to *Kurtén et al*. [2011], the reaction rate between SA**·**DMA and the CIMS reagent ion (NO_3_^−^) could be considerably slower than the reaction rate between the bare H_2_SO_4_ monomer and the nitrate ion based on quantum chemistry computations of proton transfer energies. On the other hand, a more recent quantum chemistry study by *Kupiainen‐Määttä et al*. [2013] which included different charging efficiencies of the cluster and the monomer suggested that both the bare sulfuric acid and the SA**·**DMA cluster can be ionized by the nitrate ion at the collision limit. However, due to the higher dipole moment of SA**·**DMA compared to H_2_SO_4_, the collision rate with the reagent ion could be even somewhat higher for the sulfuric acid amine cluster. Clustering of the sulfuric acid with amines would therefore tend to increase their detection probability, instead of decreasing it, as it was suggested previously. As the SA**·**DMA clusters will participate in the nucleation even more efficiently than the bare H_2_SO_4_ molecules [*Kürten et al*., 2014], it is desirable to measure the total sulfuric acid monomer concentration (i.e., the sum of H_2_SO_4_ and SA**·**DMA).

Here we define SA monomers as either the single H_2_SO_4_ molecule or clusters containing 1 SA but potentially also water molecules or amine molecules, with the term amine in this study being used for dimethylamine. Similarly, the dimer contains 2 SA molecules, the trimer 3 SA, etc., but with varying amounts of water or amine. A recent study by *Neitola et al*. [2015] showed a large discrepancy between total sulfate and sulfuric acid monomer measurements, indicating that a nonnegligible fraction of the monomer sulfuric acid was accumulated in the larger clusters. In fact, for the ternary H_2_SO_4_‐H_2_O‐(CH_3_)_2_NH system investigated at CLOUD, it was found that the measured particle growth rates exceeded the expected growth rate due to condensation of sulfuric acid monomers by about a factor of 10 (K. Lehtipalo et al., The effect of acid‐base clustering and ions on the growth of atmospheric nano‐particles, submitted to *Nature Communications*, 2015). This strongly enhanced growth could, however, not be explained merely by the presence of SA**·**DMA but also required a substantial amount of sulfuric acid contained in clusters (dimers and larger) contributing to the growth.

Based on the findings by K. Lehtipalo et al. (submitted manuscript, 2015) and the earlier work by *Kürten et al*. [2014] this study focuses on a detailed investigation of sulfuric acid monomers versus total sulfuric acid in the ternary system involving amine. In this study we compared the sulfuric acid monomer measurements of a quadrupole CIMS with the measurements made by a newly developed Chemical Ionization‐Atmospheric Pressure interface‐Time Of Flight (CI‐APi‐TOF) mass spectrometer. This comparison was made while amine was added to the CLOUD chamber during an experiment which was started as a binary nucleation experiment. From the observed signals as well as from modeling studies, conclusions regarding the sensitivity of the chemical ionization method toward sulfuric acid under the presence of amine are drawn. In addition, the importance of total sulfuric acid versus sulfuric acid monomer concentration is discussed in terms of the neutral clusters measured by the CI‐APi‐TOF.

## 2 Methods

This study presents measurements that were conducted in the CLOUD chamber at CERN between October and December 2012 (CLOUD7 campaign).

### 2.1 CLOUD Chamber

Nucleation experiments under atmospherically relevant conditions were conducted at the CLOUD (Cosmics Leaving OUtdoor Droplets) aerosol chamber. The CLOUD chamber is an electropolished stainless steel, 26.1 m^3^ chamber with unique features [*Kirkby et al*., 2011; *Duplissy et al.*, 2016]. In order to achieve minimal contamination levels the chamber and the gas system are made of stainless steel. Synthetic air is generated from cryogenic nitrogen and oxygen, which can be humidified with ultraclean water, whereas the trace gases (O_3_, SO_2_, and amine) are introduced via independent gas lines. Typical condition of the chamber is at a pressure slightly higher than ambient atmospheric pressure, at a constant temperature of 278.15 K and at a relative humidity (RH) of 38%. The homogeneous distribution of chamber air is established by a pair of mixing fans [*Voigtländer et al*., 2012] while its irradiation is achieved by means of a unique UV fiber optic system [*Kupc et al*., 2011]. The intensity of the provided UV light can be adjusted by means of an aperture. The UV light drives photochemical reactions inside the chamber, most prominently the generation of OH radicals in the presence of ozone and water vapor. Gaseous H_2_SO_4_ is then formed in situ from the reaction of SO_2_ with OH [*Kupc et al*., 2011].

A focus of the CLOUD measurements is to distinguish aerosol nucleation processes initiated with and without the aid of charged molecules. The CERN Proton Synchrotron can be used to expose the CLOUD chamber to a diverged beam of 3.5 GeV/c pions (π^+^), creating variable levels of ion concentrations in the chamber [*Duplissy et al*., 2010]. Therefore, three different modes of operation are realized in the nucleation experiments: (a) neutral mode (n) where all ion‐induced nucleation in the chamber is suppressed by application of an electric field with a potential difference of 60 kV across the chamber which sweeps out all ions within less than 1 s; (b) natural charged mode (GCR) when the electric clearing field is switched off, where ions that are produced from natural galactic cosmic rays can initiate ion‐induced nucleation; and (c) pion beam charged mode (ch) when the pion beam from the Proton Synchrotron is switched on and the clearing field is switched off, where ionization in the chamber can be increased by up to a factor of 10 or more compared to GCR conditions in order to simulate ionizing conditions relevant to the upper troposphere. Therefore, the influence of ions on nucleation can be investigated in detail [*Kirkby et al*., 2011]. However, for the present study involving sulfuric acid, water, and amine, neutral nucleation dominates by far [*Almeida et al*., 2013; *Kürten et al*., 2014]; therefore, no distinction between neutral and ion‐induced conditions is made in the following. During the CLOUD7 campaign, a suite of instruments was connected to the chamber including several condensation particle counters (CPCs), mass spectrometers, and electrical mobility analyzers. Additionally, an ion chromatograph [*Praplan et al*., 2012] was used to determine the mixing ratio of dimethylamine and ammonia.

### 2.2 CIMS Measuring Technique

Chemical Ionization Mass Spectrometer (CIMS) is widely used both in laboratory and field experiments for the very sensitive detection of gaseous H_2_SO_4_ down to parts per quadrillion by volume levels in real time [*Eisele and Tanner*, 1993; *Berresheim et al*., 2000; *Petäjä et al*., 2009]. A CIMS system (THS Instruments LLC, USA) is used to measure the concentration of gaseous H_2_SO_4_ during all CLOUD campaigns [*Kürten et al*., 2011] at a detection limit of ~1 × 10^5^ molecule cm^−3^. Just before and after the campaign, a known concentration of sulfuric acid was produced by a stand‐alone calibration system in order to ensure highly accurate and reproducible measurements [*Kürten et al*., 2012]. The calibration system produces a known and stable concentration of sulfuric acid providing a calibration factor of 1.1 × 10^10^ molecule cm^−3^ that relates the measured ion signals to a true sulfuric acid concentration derived from a numerical model.

The working principle of the CIMS is to selectively charge sulfuric acid in the sample gas by means of chemical ionization and then detect the product ions with a mass spectrometer. A small fraction of the H_2_SO_4_ is thereby converted to bisulfate (HSO_4_^−^) through the reaction with nitrate primary ions (NO_3_^−^(HNO_3_)_*k* = 0–2_). These reagent ions are produced from a negative corona discharge when nitric acid is added to the sheath gas of the CI part of the instrument [*Kürten et al*., 2011; *Rondo et al*., 2014]. Using this method, it is thought that both the bare sulfuric acid molecules as well as the SA**·**DMA clusters produced in the CLOUD chamber are ionized via the following reactions:

(R1) $$H_{2}{\text{SO}}_{4}+{{\text{NO}}_{3}}^{–}{\left({HNO}_{3}\right)}_{k}\to \;{{HSO}_{4}}^{–}{\left({HNO}_{3}\right)}_{y}+\;\left(k-y+1\right)\cdot \left({HNO}_{3}\right)$$

(R2) $$H_{2}{\text{SO}}_{4}•DMA\;+\;{{\text{NO}}_{3}}^{–}{\left({HNO}_{3}\right)}_{k}\to \;{{HSO}_{4}}^{–}{\left({HNO}_{3}\right)}_{y}+\;\left(k-y+1\right)\cdot \left({HNO}_{3}\right)\;+\;DMA$$

All species of reaction (R1) and (R2) can also be clustered with variable amounts of water molecules, but this is omitted here for simplicity. Reaction (R2) indicates that the base (amine) rapidly evaporates after the SA**·**DMA cluster is ionized [*Ortega et al*., 2014]. In addition, the ionized clusters can undergo fragmentation in a collision dissociation chamber (CDC) by energetic collisions with neutral molecules, where most sulfuric acid monomer ions are converted into HSO_4_^−^, and most reagent ions are likewise converted to NO_3_^−^. While we expect that DMA already evaporates from the sulfuric acid monomer ions in the flow tube, due to the use of the CDC, any remaining DMA will be stripped off and any direct detection of DMA associated with a sulfuric acid monomer is not possible. Since the sulfuric acid monomer concentration is evaluated from the count rates of HSO_4_^−^ and NO_3_^−^, it includes the contribution from H_2_SO_4_ as well as from SA**·**DMA. Regarding the charging efficiency of the sampled SA**·**DMA clusters, there is yet no experimental evidence but only theoretical studies supporting either lower [*Kurtén et al*., 2011] or higher charging efficiency [*Kupiainen‐Määttä et al*., 2013] compared with the bare H_2_SO_4_ molecules. However, if the second reaction (R2) proceeded at a significantly different rate compared to (R1) an error would be introduced in evaluating the concentration of the total sulfuric acid monomer using the CIMS calibration factor, which is derived for reaction (R1). The first injection of amine during the CLOUD7 campaign, starting from the cleanest possible conditions with background concentration of ammonia and DMA less than 35 pptv and less than 0.2 pptv, respectively, was therefore performed while sulfuric acid was photolytically produced at a constant rate. In this way we could study if the observed signal of HSO_4_^−^ would change significantly upon the amine injection.

### 2.3 CI‐APi‐TOF Measuring Technique

A detailed insight into the elemental composition of molecular clusters in the sulfuric acid, water, and amine system is provided by a Chemical Ionization‐Atmospheric Pressure interface‐Time of Flight (CI‐APi‐TOF) mass spectrometer [*Kürten et al*., 2014]. The CI‐APi‐TOF is a state‐of‐the‐art instrument which uses a similar chemical ionization scheme as the CIMS, i.e., by nitrate ions [*Jokinen et al*., 2012; *Kürten et al*., 2014]. However, compared to the CIMS the CI‐APi‐TOF has several advantages because it uses a time‐of‐flight mass spectrometer (TOF‐MS) instead of a quadrupole as the mass analyzer. The TOF‐MS used in this study reaches a mass resolving power up to ~4500 Th/Th while achieving a mass accuracy of better than 10 ppm. These features, together with the measured isotopic patterns, allow retrieval of the exact elemental composition of neutral clusters. The mass range can easily reach up to 2000 Th. For the SA‐DMA system neutral clusters containing as many as 14 SA and 16 DMA molecules were observed at CLOUD with some of the smallest sulfuric acid molecules (up to tetramer) detected without any DMA attached due to competition with the other Lewis bases, HSO_4_^−^ and NO_3_^−^ [*Kürten et al*., 2014]. In case that some of the HNO_3_ does not evaporate from the newly formed charged cluster, the DMA would be stabilized in the SA**·**DMA cluster as shown via the following reactions:

(R3) $${\left(H_{2}{\text{SO}}_{4}\right)}_{1-4}•DMA\;+\;{{\text{NO}}_{3}}^{–}{\left({HNO}_{3}\right)}_{k}\to \;{{HSO}_{4}}^{–}{\left(H_{2}{\text{SO}}_{4}\right)}_{0-3}{\left({HNO}_{3}\right)}_{y=0}+\;\left(k-y+1\right)\cdot \left({HNO}_{3}\right)\;+\;DMA$$

(R4) $${\left(H_{2}{\text{SO}}_{4}\right)}_{i>1}•DMA\;+\;{{\text{NO}}_{3}}^{–}{\left({HNO}_{3}\right)}_{k}\to \;{{HSO}_{4}}^{–}{\left(H_{2}{\text{SO}}_{4}\right)}_{i-1}DMA\;{\left({HNO}_{3}\right)}_{y>0}+\;\left(k-y+1\right)\cdot \left({HNO}_{3}\right)$$

In our study monomer sulfuric acid (N_1_), dimer (N_2_), trimer (N_3_), tetramer (N_4_), and pentamer (N_5_) refer to the number of sulfuric acid molecules in the cluster coupled with H_2_O and amine molecules. To obtain the cluster concentrations (N_2_ to N_5_), the signals with different amounts of amine but with a certain number of sulfur atoms are added up [*Kürten et al*., 2014]. Furthermore, detection limits below 10^4^ cm^−3^ can be reached for clusters containing four or five sulfuric acid and several amine molecules [*Kürten et al*., 2014]. In this study, two CI‐APi‐TOFs were used from the Universities of Frankfurt and Helsinki. The differences and similarities of the two instruments are described elsewhere [*Kürten et al*., 2014]. Main differences between the two CI‐APi‐TOF instruments include different ionization sources (soft X‐ray and corona discharge), the introduction of an ion precipitator in the sampling line (included only in CI‐APi‐TOF‐Frankfurt), and different geometries of the ion‐molecule reactor resulting in different reaction times.

Similar to the CIMS, the CI‐APi‐TOFs are also not capable of distinguishing between bare H_2_SO_4_ and SA**·**DMA. In order to evaluate the effect of amine on the sulfuric acid monomer concentration the CI‐APi‐TOFs are nevertheless very important. When amine is added to the chamber while sulfuric acid is produced at a constant rate, the CI‐APi‐TOFs can indicate whether higher‐order clusters containing sulfuric acid and amine are produced at a significant rate. If this process occurs, it represents a loss channel for the monomers, which would need to be taken into account when the monomer concentration and the production rate are analyzed.

### 2.4 Modeled Sulfuric Acid Molecules and Clusters

#### 2.4.1 Modeled Sulfuric Acid Concentrations Using ASAD

The ASAD (A Self‐contained Atmospheric chemistry coDe) model for atmospheric chemistry [*Carver et al*., 1997] was adapted to model the sulfuric acid production in the CLOUD chamber. Chemical tracers that represent the sulfuric acid gas phase oxidation in a system of SO_2_, O_3_, and H_2_O vapor in air are displayed in Table S1 in the supporting information. Organic compounds or amine were not included in the chemical scheme mainly because their contribution to OH chemistry in the system was considered minimal. The adjustments made for the extended use in CLOUD chamber conditions include the introduction of wall losses, the photolysis rates due to the UV light system of the CLOUD chamber, and losses to the aerosol particles. The wall loss rate constant, *k*_i_, for a species *i* is described by diffusional wall losses as shown by [*Crump and Seinfeld*, 1981; *Metzger et al*., 2010]

(1) $$k_{i}=C\times \sqrt{D_{i}}\text{.}$$

*C* is determined so that the wall loss of H_2_SO_4_ agrees with the wall loss rate constant of 1.7 × 10^−3^ s^−1^ given in *Almeida et al*. [2013], while the diffusion coefficient *D* of H_2_SO_4_ is taken from *Hanson and Eisele* [2000]. Diffusion coefficients, *D_i_*, of other small chemical species (such as OH, H_2_O_2_, HO_2_) are estimated from kinetic gas theory. The trace gases O_3_, SO_2_, and H_2_O are considered to be in equilibrium with the wall.

The UV light photolysis rate, *j_i_*, is implemented as function of the UV light aperture and is proportional to the UV light power as given in *Kupc et al*. [2011]

(2) $$j_{i}=a\times P_{ap}\times j_{th}\text{.}$$

The theoretical UV light photolysis rate, *j*_th_, for production of O(^1^D) and O(^3^P) from O_3_ as well as the photolysis of H_2_O_2_ to OH was calculated based on the UV light spectrum while factor *a* is a parameter to adjust the photolysis rate to the UV light in the chamber. The necessity of this factor relies on the fact that the actual photon flux in the chamber is not known and has to be determined for each CLOUD campaign due to changes in the UV lamps, such as aging or replacement. For this study the photolysis rate was adjusted based on fits to experiments without any amine present. The polynomial relationship between UV aperture (per cent) and UV intensity (per cent) represented as *P*_ap_ is defined in *Kupc et al*. [2011] and translates the relative aperture opening in per cent into a relative UV power output. The absolute photolysis rates, in per second, at fully opened UV aperture for the production of O(^1^D) and O(^3^P) and OH are 2.3 × 10^−6^, 2.7 × 10^−7^, and 2.5 × 10^−8^, respectively.

#### 2.4.2 SAWNUC Model

The SAWNUC (Sulfuric Acid Water NUCleation) model was originally developed for binary sulfuric acid‐water nucleation [*Lovejoy et al*., 2004]. It has been modified to be applied for effectively one‐component cluster formation proceeding at the kinetic limit when amine is present by removing the evaporation rate and defining the density and composition of the monomer to correspond to the SA**·**DMA cluster [*Ehrhart and Curtius*, 2013; *Kürten et al*., 2014]. Sulfuric acid processes within the CLOUD chamber such as condensation, coagulation, and losses due to walls and dilution are included in the model [*Ehrhart and Curtius*, 2013]. A generic monomer with a composition of one H_2_SO_4_ and one amine was used for the simulations; the ratio of 1:1 was also assumed for the clusters and particles. The density of H_2_SO_4_‐DMA was set to 1400 kg m^−3^ [*Qiu and Zhang*, 2012]. In order to model the amount of sulfuric acid in the small aerosol clusters the ASAD model and a modified version of SAWNUC were combined to provide a time series of sulfuric acid and cluster concentrations. The concentration of sulfuric acid monomers is calculated from ASAD using the concentrations of SO_2_, O_3_, and H_2_O, the temperature of the chamber, and the UV aperture of CLOUD's UV system. In Figure SI of the supporting information, a scheme of the information flow revealing the steps required for the sulfuric acid evolution is displayed. Based on these assumptions SAWNUC then calculates the cluster evolution and determines the additional sinks due to condensation of monomers onto the particles and cluster.

Wall loss of clusters was implemented in the same way as for H_2_SO_4_, but diffusion coefficients were calculated from Cunningham‐corrected Stokes‐Einstein diffusion coefficients [*Baron and Willeke*, 2001].

## 3 Results

### 3.1 Sulfuric Acid Monomer Measurements During Amine Introduction Into the CLOUD Chamber

**Figure 1:**
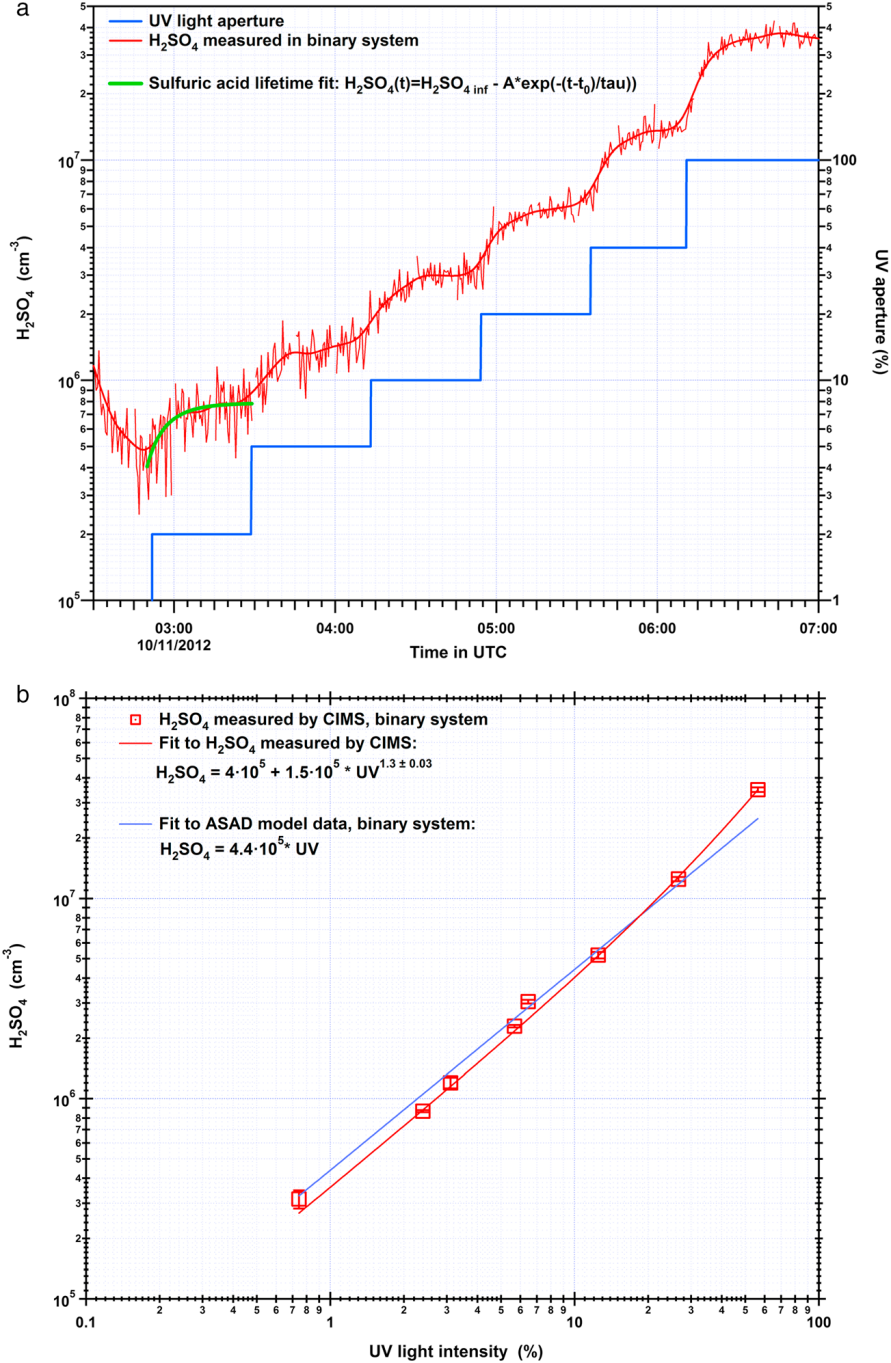
(a) Example of a measurement of the sulfuric acid concentration (displayed with red color for both 5 s raw data and 1 min averaged data) a variation of the UV light (10% to 100% UV light aperture) for the pure H_2_SO_4_‐H_2_O binary system. (b) Comparison of the expected H_2_SO_4_ (ASAD model and UV light dependency) and the average measured H_2_SO_4_ concentration for different UV light intensities applied in the chamber after equilibration of the H_2_SO_4_ concentration to the new settings. The displayed CIMS sulfuric acid concentrations include 1σ total errors, while the systematic scale uncertainty is a factor of 2. The averaged measured concentrations are taken after H_2_SO_4_ equilibrium is established within 650 ± 33 s, which is given from the sulfuric acid lifetime fit as shown in Figure 1a.

In the present study the effect of dimethylamine on the derived sulfuric acid concentration is investigated from data obtained during nucleation experiments, where the sulfuric acid monomer and its clusters were measured under varying conditions. Initially, experiments were performed for the pure binary H_2_SO_4_‐H_2_O system with other condensable compounds present only as trace impurities [*Bianchi et al*., 2012]. A range of H_2_SO_4_ concentrations from 1 × 10^6^ to 1 × 10^8^ molecule cm^−3^ was studied. The production of gaseous sulfuric acid is established by means of photolytic reactions of the trace gases O_3_ and SO_2_ initiated by the UV light illumination. Variation of the UV light intensity results in a proportional response in the sulfuric acid production rate, with its equilibrium concentration being established within 650 ± 33 s when the production rate is balanced by the wall loss rate. An overview of a typical binary experimental run is given in Figure 1a, which displays the evolution of the H_2_SO_4_ concentration for different UV light aperture settings. The measured sulfuric acid monomer concentrations show an almost linear dependence on the applied UV light intensity for the binary system (Figure 1b). This observation is in agreement with an earlier CLOUD study where the dependence of the measured sulfuric acid on the UV intensity and aperture settings was investigated [*Kupc et al*., 2011]. It was shown to be nearly linear for low values, while for larger values it can be approximated by a third‐order polynomial fit [*Kupc et al*., 2011]. In our present study a chemical model (ASAD) was applied where UV light intensity, RH, O_3_, and SO_2_ gas concentrations were included. A comparison of the sulfuric acid monomer concentrations experimentally measured and calculated with the ASAD model shows good agreement (Figure 1b).

**Figure 2:**
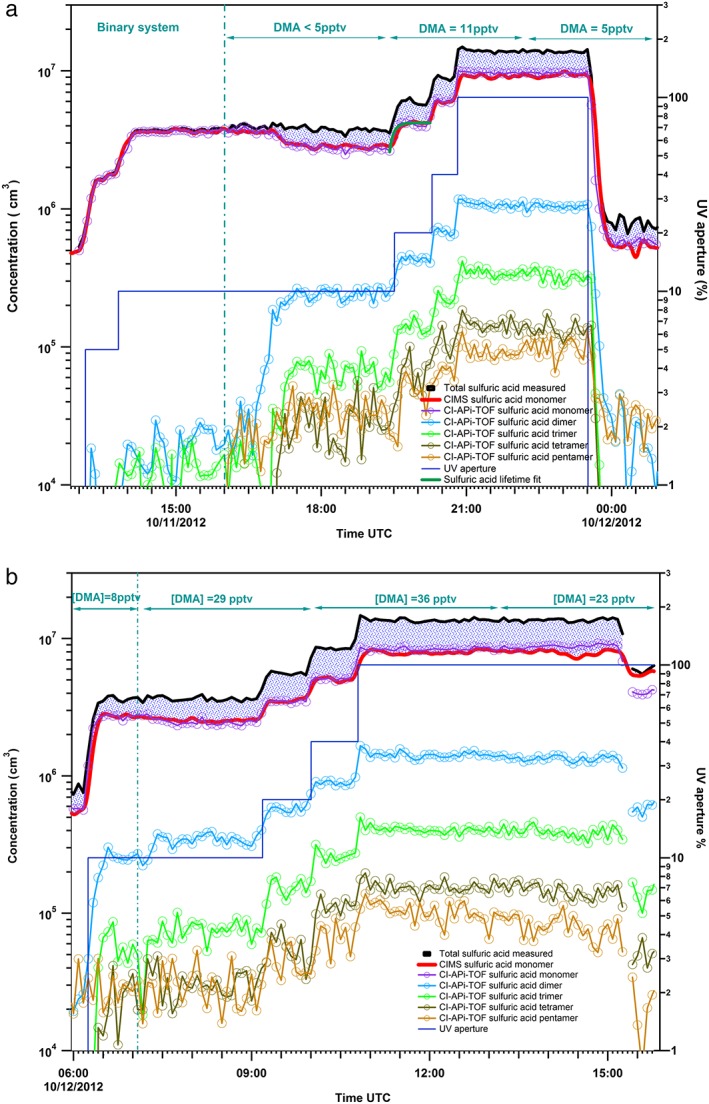
(a) Sulfuric acid monomer and cluster concentrations containing 2 to 5 H_2_SO_4_ molecules as measured by CIMS and CI‐APi‐TOF mass spectrometers for different UV light aperture settings. The total sulfuric acid concentration derived from the sum of the measured sulfuric acid monomers and clusters up to the pentamer is also displayed. The difference between the sulfuric acid monomer and the total sulfuric acid concentration indicates the amount of the H_2_SO_4_ shifted from the monomer into the larger clusters (blue shaded area). For the amine‐ternary system the sulfuric acid lifetime fit results to 150 ± 60 s as averaged time required for the H_2_SO_4_ monomer concentration to reach equilibrium. (b) Sulfuric acid measurements when increasing the dimethylamine concentration to 36 pptv in the CLOUD chamber demonstrating some additional shift of sulfuric acid monomers into larger clusters.

To study the influence of amine on the quantitative detection of the H_2_SO_4_ monomer by the CIMS, the amine (~11 pptv) was added to the CLOUD chamber after equilibrium of the sulfuric acid concentration was reached for binary conditions (Figure 2a). In this way potential influences of the selected base, amine, on the measured sulfuric acid monomer, and its clusters can be detected in a very direct way by the chemical ionization mass spectrometers. Note that after opening the valves and introducing amine into the chamber it took about 2 h for the amine to be detected inside the chamber. Indeed, within these 2 h the sulfuric acid monomer concentration was significantly reduced with respect to the prior measurement of the binary system (Figure 2a). The CIMS and the CI‐APi‐TOF measurements showed that the introduction of ~5 pptv of dimethylamine into the system leads to a decrease of 23% in the sulfuric acid monomer signal (*m*/*z* 97) (Figure 2a), while additional amine introduction (~36 pptv) resulted in an additional 7% decrease of the H_2_SO_4_ monomer signal (Figure 2b).

**Figure 3:**
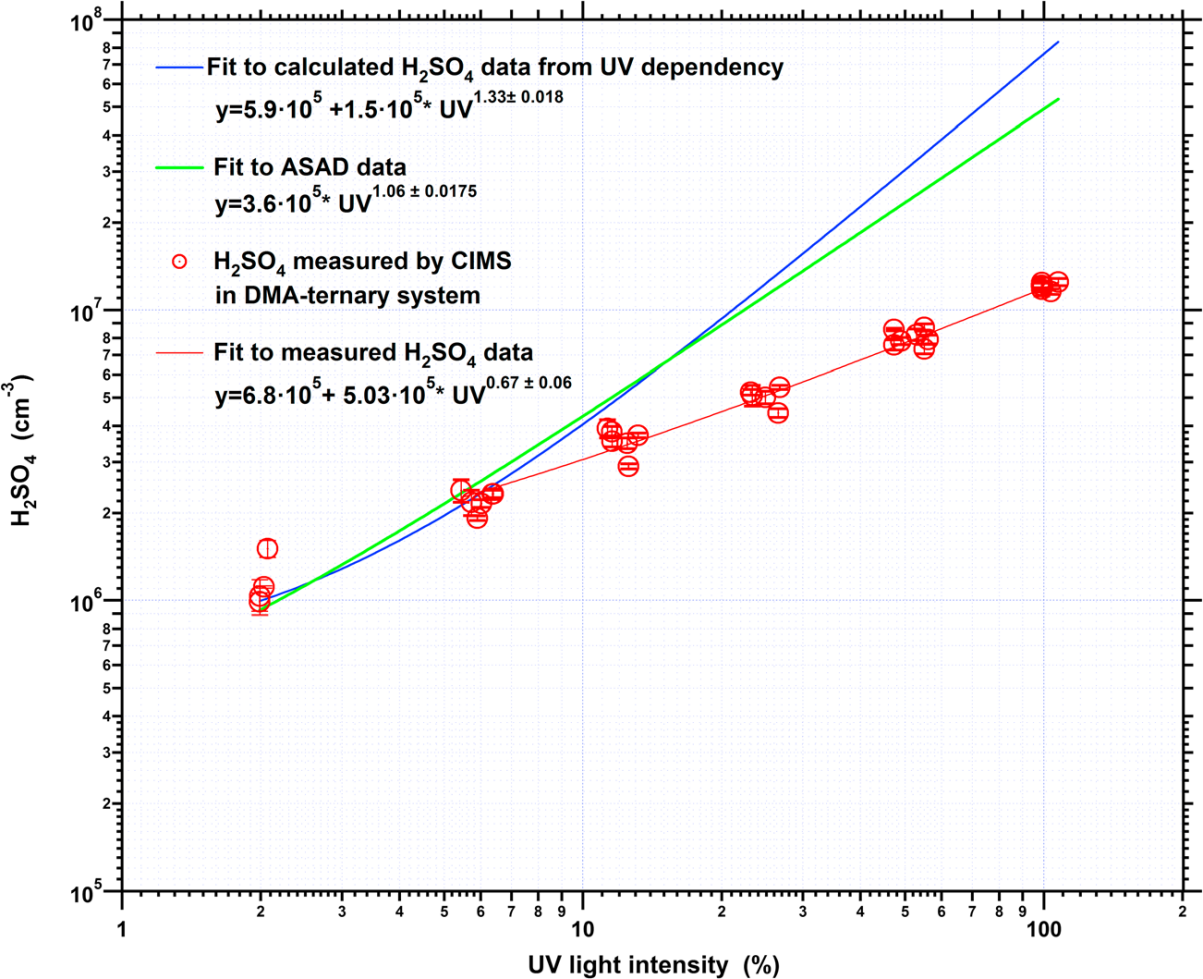
Comparison of the expected monomer H_2_SO_4_ (ASAD model, green colored, and the UV light dependency, blue colored) for the pure binary system, and the measured H_2_SO_4_ monomer concentration for the amine‐ternary system (red) as a function of UV light intensity. Displayed data points represent all experimental runs of the amine‐ternary system.

Apart from the decrease of the measured monomer sulfuric acid concentrations, we also observed a change of the characteristic time period of the H_2_SO_4_ monomer signal to reach steady state. As shown in Figure 2a, the time required for the H_2_SO_4_ monomer concentration to reach steady state is about a factor of 4 lower compared to the binary system. This observation is explained by the fact that while the production rate of sulfuric acid monomers remained constant, its concentration is being affected by the presence of the amine in the system due to a change in the losses. As a result, the linear dependency of the produced sulfuric acid monomers to the applied UV light intensity as derived for the binary system no longer applies to the amine ternary system. The produced sulfuric acid monomer concentration deviates significantly from the expected linear dependency on the UV light intensity, indicating a close to square root dependency (Figure 3). The discrepancy between the expected and the measured sulfuric acid concentration at the higher UV light intensities indicates that a significant part of the produced H_2_SO_4_ monomers are transferred into the larger clusters due to the efficient amine stabilization effect preventing evaporation of the clusters. The loss of sulfuric acid monomers is in this case dominated by self coagulation between two monomers, which can efficiently form a dimer if at least one of the monomers is present as SA**·**DMA [*Kürten et al*., 2014]. The sulfuric acid monomer concentration establishes its equilibrium faster due to the fact that a significant fraction of H_2_SO_4_ is consumed by processes other than those governing the binary system (losses due to walls, dilution, and condensation onto preexisting large aerosol particles, but only insignificant cluster formation).

Regarding the alteration of the sulfuric acid‐water system due to the introduction of amine, we should take into consideration the potential contribution of several other effects in addition to the formation of the larger clusters: change of diffusivity of the monomers, OH consumption, or any change of the ion‐molecule‐reaction rate in the CIMS‐reactor (*k*_R1_ ≠ *k*_R2_) [*Kurtén et al*., 2011; *Kupiainen‐Määttä et al*., 2013], in addition to the formation of the larger clusters. The addition of an amine molecule to the sulfuric acid monomer changes its diffusivity. This will have an effect both on the wall loss rate and on the sampling line transmission to the mass spectrometers. Below 38% RH the sulfuric acid monomer will be bound to one or two water molecules [*Hanson and Eisele*, 2000]. It is not exactly known what happens to the water if an amine molecule attaches to the hydrated sulfuric acid, but theoretical studies report that SA**·**DMA cluster can bind to water molecules and its effective evaporation rate decreases [*Olenius et al*., 2014]. Therefore, the sulfuric acid associated with an amine should have a somewhat reduced diffusivity compared with the sulfuric acid in the binary system. Since the wall loss rate of a molecule or cluster is proportional to the square root of its diffusivity, the concentration of sulfuric acid in the ternary amine system should increase slightly for a given production rate. In addition, the sampling line losses should be somewhat reduced. We cannot fully quantify this effect, but we assume that it is minor. Another effect that influences the production of sulfuric acid during the main experiment is that OH radicals, which lead to the production of H_2_SO_4_ from reaction with SO_2_, are partly consumed due to the added amine. The reaction rate constant between OH and dimethylamine (6.5 × 10^−11^ cm^3^ molecule^−1^ s^−1^ at 298 K) is approximately 7 times larger than the one between OH and SO_2_ {9 × 10^−12^ cm^3^ molecule^−1^ s^−1^ at 298 K [*Atkinson et al*., 2004]). However, the mixing ratio of SO_2_ during CLOUD7 was 60 ppbv compared to a maximum of ~70 pptv of amine. Therefore, the expected change in the produced H_2_SO_4_ due to the amine addition should be less than 0.5%. The possibility of a significant difference of the ion molecule reaction rate constants *k*_R1_ and *k*_R2_ will be discussed below.

### 3.2 Sulfuric Acid Cluster Measurements by CI‐APi‐TOF

While the measurements show a decrease of the sulfuric acid monomer concentration, the CI‐APi‐TOF's cluster measurements reveal information on cluster formation that explains the observed reduction of the monomer to a large degree. As shown in Figure 2a, the sulfuric acid‐amine clusters (dimer to pentamer) increased when the dimethylamine was introduced into the chamber while the sulfuric acid monomer concentration simultaneously decreased. This observation implies that a significant fraction (~30%) of the produced H_2_SO_4_ monomer is being transferred into larger SA*_x_*DMA*_y_* (*x* > 1) clusters that do not evaporate rapidly, confirming the stabilizing role of the amine. A recent study by *Kürten et al*. [2014] reported detection of clusters containing up to 14 H_2_SO_4_ and 16 DMA molecules confirming the important role of amines for the formation of stable clusters by nucleation with sulfuric acid. In addition, as displayed in Figure 2b, further addition of amine (leading to a total mixing ratio of ~36 pptv) resulted in further increases of the sulfuric‐acid‐containing clusters (dimer to pentamer).

**Figure 4:**
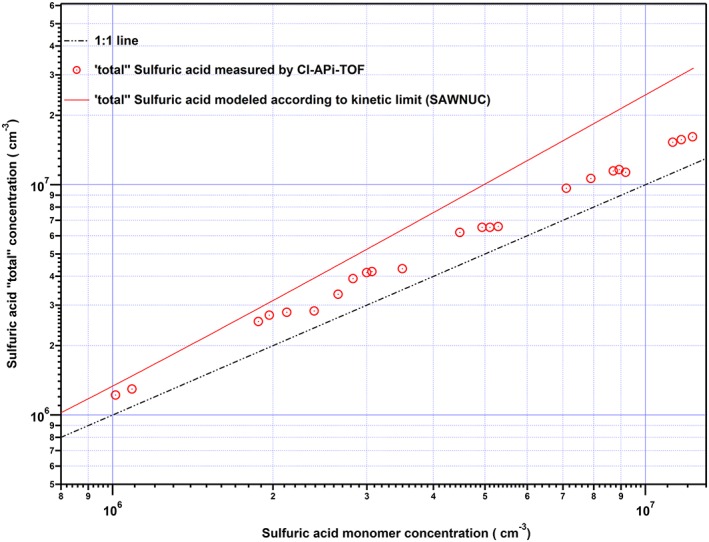
Total sulfuric acid concentration in comparison to the H_2_SO_4_ monomer measurements during the amine‐ternary nucleation experiments. From the measured total sulfuric acid concentrations (red circles), the monomer concentration (1:1 line) deviates by a factor 1.2 to 1.6, indicating the shift of a fraction of monomers into larger clusters. However, the modeled total (up to pentamer) sulfuric acid concentration according to the kinetic limit assumption (red curve) reveals that there is still an amount of sulfuric acid which is not measured due to possible decreasing transmission and charging efficiencies of the CI‐APi‐TOF.

We derived a quantity that we call *total sulfuric acid*, SA_total_ by summing up the SA molecules contained in the monomers and clusters up to the pentamer; *SA_total_* = $\sum_{i=1}^{5}i$ *N_i_* (Figures 2 and 4). Evaluation of the sulfuric acid cluster concentration *N_i_* has been described in full detail by the study of *Kürten et al*. [2014]. In general, for the concentration of the monomer *N_1_* a calibration constant derived from the calibration of the CI‐APi‐TOFs (according to the method described by *Kürten et al*. [2012]) along with a transmission efficiency factor is taken into account. As for the evaluation of the higher sulfuric acid clusters *N_i_*, so far there is no direct calibration of these clusters. Thus, we include additional corrections including the different reaction rates between cluster and primary ions compared to the monomer, the transmission efficiency, and mass discrimination effects in the CI‐APi‐TOF.

Although there are even larger clusters, we define the total sulfuric acid in this study as the sulfuric acid which is contained in clusters up to the pentamer because this was the largest size for which the concentrations were quantitatively derived [*Kürten et al*., 2014]. For the binary system, the sulfuric acid monomer concentration agrees well with the total sulfuric acid concentration, whereas for the amine‐ternary system the discrepancy increases. Total sulfuric acid data representing all the performed amine‐ternary experimental runs are displayed in Figure 4. The comparison of the measured total to the monomer sulfuric acid concentration shows a shift of the available sulfuric acid monomer concentration into the stabilized larger clusters changing the monomer/total ratio by a factor of 1.2–1.6.

**Figure 5:**
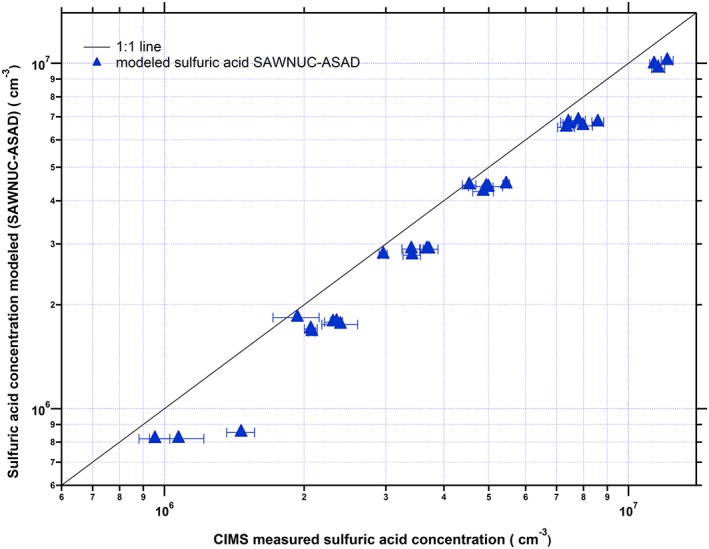
Comparison of the modeled monomer sulfuric acid concentration (SAWNUC kinetic limit model in combination with ASAD) and the measured monomer sulfuric acid concentration during amine‐ternary nucleation experiments. The displayed results indicate a good agreement between model predictions and the CIMS measured sulfuric acid monomer concentration suggesting this way that CIMS detection efficiency is not significantly affected by the presence of dimethylamine.

In order to investigate quantitatively the magnitude of the “hidden” sulfuric acid that contributes to coagulation and growth but is not measured by the CIMS, our experimental measurements were compared with theoretical predictions (Figure 4). All possible collisions and coagulation are explicitly simulated in a kinetic model, SAWNUC, in order to describe the cluster distribution for a certain set of clusters (up to the pentamer). Assuming the kinetic limit, any collision between an SA *i*‐mer with another SA *i*‐mer leads to a further clustering while it is assumed that no evaporation of clusters occurs. Indeed, the comparison of the modeled (red curve) and measured total sulfuric acid cluster concentration (red dots) reveals that there is still a discrepancy between the measured total sulfuric acid and the amount expected for the kinetic limit. The discrepancy between the modeled and measured total sulfuric acid indicates either a declining transmission of the mass spectrometer for larger clusters or a decrease of the ion‐molecule reaction rate constants with increasing cluster size. Most likely, these effects can explain the remaining discrepancy between the kinetic limit calculation and the observed total sulfuric acid. Regarding the magnitude of the discrepancy between total sulfuric acid and sulfuric acid monomer, it is also important to note that it can be even larger than what is shown in Figure 4 if clusters larger than the pentamer are taken into account. In addition, a scatterplot for the resulting modeled and the experimentally observed equilibrium sulfuric acid monomer concentrations are displayed in Figure 5. The good agreement confirms that the CIMS sulfuric acid monomer detection efficiency is not significantly affected by the presence of amine. Nevertheless, note that the true impact of the stabilized SA**·**DMA cluster in the atmosphere is a function of the conditions and will depend on the cluster distribution under specific conditions, taking into consideration, for example, losses to preexisting particles. A more detailed study regarding the contribution of clustering to the nanoparticle growth over a wide range of atmospheric conditions is included in the submitted study of K. Lehtipalo et al. (submitted manuscript, 2015).

## 4 Summary and Conclusions

The capability of sulfuric acid molecules to bind with base molecules such as ammonia (NH_3_) and dimethylamine (DMA) leading to the formation of larger clusters and particles has been demonstrated in earlier studies [*Kirkby et al*., 2011; *Almeida et al*., 2013; *Bianchi et al*., 2014; *Kürten et al*., 2014]. In comparison to the H_2_SO_4_‐H_2_O‐NH_3_ system, the nature of the chemical bond of sulfuric acid to dimethylamine reveals that the H_2_SO_4_‐H_2_O‐DMA system forms structures that are significantly more strongly bound [*Loukonen et al*., 2010], leading to particle formation at (or close to) the kinetic limit for parts per trillion by volume levels of DMA [*Kürten et al*., 2014]. Chemical ionization mass spectrometric (CIMS) techniques are used for the quantification of the H_2_SO_4_ monomer concentrations in the atmosphere and in the laboratory. It has been suspected that the efficiency of the ion‐molecule‐reaction that is used for the detection of the H_2_SO_4_ monomers changes in the presence of dimethylamine [*Kurtén et al*., 2008; *Kupiainen‐Määttä et al*., 2013]. In the present experimental study we investigated the mass spectrometric signals of H_2_SO_4_ monomers in the absence and presence of dimethylamine to see if the monomer signals change either (a) due to the onset of highly efficient particle nucleation and formation of clusters which reduce the monomer signal or (b) because the rate constant *k*_R2_ significantly differs from *k*_R1_.

H_2_SO_4_ monomers and freshly nucleated molecular clusters were produced photochemically under well‐controlled conditions in the CLOUD chamber and analyzed with CIMS and a newly developed mass spectrometer (CI‐APi‐TOF). Distinct differences were observed, from both CIMS and CI‐APi‐TOF, between binary H_2_SO_4_‐H_2_O and ternary H_2_SO_4_‐H_2_O‐DMA nucleation experiments. It was found that for the H_2_SO_4_‐H_2_O‐DMA ternary system, the sulfuric acid monomer concentration as measured by the bisulfate ion (*m*/*z* 97) was influenced by the presence of dimethylamine. We measured monomer sulfuric acid concentration using both CIMS and CI‐APi‐TOF mass spectrometers and observed a reduction of the monomer signal after DMA was introduced. In addition, a simultaneous increase of all the larger sulfuric acid clusters (dimer to pentamer) was observed. Therefore, the linear dependency of the monomer sulfuric acid to the UV light intensity that applies to the binary system does not hold for the amine ternary system anymore. Instead, a close to square root dependency is observed. From the binary and DMA ternary experiments it was found that the time required for the H_2_SO_4_ monomer concentration to reach its equilibrium is lowered in the presence of amine by a factor of ~4, although the production rate of sulfuric acid remained constant. This is explained by the increased monomer sink due to efficient formation of dimers and collisions with larger clusters. It is suggested that effects such as changing diffusivity of the clusters, OH consumption due to reaction with amine, and changing ion molecule reaction rate after the introduction of amine cause only a minor contribution to the change of the monomer signal. The CI‐APi‐TOF measurements do provide information on cluster composition that explains most of the observed effect.

The experimental results are in good agreement with modeling studies when assuming that the base‐stabilization by amine leads to kinetically controlled growth of the sulfuric acid clusters where every sulfuric acid *i*‐mer that collides with another sulfuric acid *i*‐mer sticks to that cluster. A remaining difference is most likely contributed to reduced transmission of the larger clusters in the CI‐APi‐TOF and potentially a smaller ion‐molecule reaction rate constant for the higher *i*‐mers compared to the monomer. In addition, the obtained results of sulfuric acid monomer and its clusters from the chemical ionization mass analyzers are in good agreement with the performed SAWNUC‐ASAD chemical model.

A major conclusion of our study is that the monomer concentrations of H_2_SO_4_ are correctly measured by the CIMS technique regardless of the presence of amine, therefore not supporting suggested changes of the charging efficiency compared with the bare H_2_SO_4_ molecules as proposed by *Kurtén et al*. [2011] or *Kupiainen‐Määttä et al*. [2013]. But the study also demonstrates that the amount of sulfuric acid contained in the monomers plus small clusters, which contributes, for example, very significantly to the growth of freshly nucleated particles as shown by K. Lehtipalo et al. (submitted manuscript, 2015), is different from the monomer sulfuric acid. It is therefore advisable for future measurements in the laboratory and in the field to distinguish carefully between the amount of monomer sulfuric acid and total sulfuric acid. In order to assess correctly the contribution of H_2_SO_4_ to the growth, ideally the dimers and larger clusters have to be measured quantitatively as well to determine the amount of sulfuric acid “hidden” in these clusters and the total sulfuric acid. Furthermore, substances like dimethylamine which are able to shift a significant fraction of the monomers into larger *i*‐mers have to be determined along with the H_2_SO_4_ measurements.

## Supporting information



Figure S1 and Table S1
