# Supplementary material for: Effect of dimethylamine on the gas phase sulfuric acid concentration measured by Chemical Ionization Mass Spectrometry
Source: J Geophys Res Atmos. 2016 Mar 24;121(6):3036–49. doi: 10.1002/2015JD023868 (PMC4996328; doi:10.1002/2015JD023868)
Supplement: Supplementary file 1 — Figure S1 and Table S1 [file JGRD-121-3036-s001.doc]

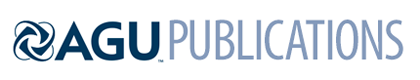


Journal of Geophysical Research - Atmospheres

Supporting Information for

**Effect of dimethylamine on the gas phase sulfuric acid concentration measured by Chemical Ionization Mass Spectrometry (CIMS)**

L.Rondo^1^, S. Ehrhart^2^, A. Kürten^1^, A. Adamov^3^, F. Bianchi^4,5^, M. Breitenlechner^6^, J. Duplissy^3^, A. Franchin^3^, J. Dommen^4^, N. M. Donahue^14^,E. M. Dunne^7,17^, R. C. Flagan^16^, J. Hakala^3^, A. Hansel^6^, H. Keskinen^3,8^, J. Kim^8^, T. Jokinen^3^, K. Lehtipalo^3^, M. Leiminger^1^, A. Praplan^3,7^, F. Riccobono^4^, M. P. Rissanen^3^, N. Sarnela^3^, S. Schobesberger^3,9^, M. Simon^1^, M. Sipilä^3^, J. N. Smith^8,10^, A. Tomé^11^, J. Tröstl^4^, G. Tsagkogeorgas^12^, P. Vaattovaara^8,15^, P. M. Winkler ^13^, C. Williamson^1^, U. Baltensperger^4^, J. Kirkby^1,2^, M. Kulmala^3^, D. R. Worsnop^3,18^ and J. Curtius^1^

^1^Institute for Atmospheric and Environmental Sciences, Goethe University Frankfurt am Main, 60438 Frankfurt am Main, Germany.

^2^CERN, CH-1211 Geneva 23, Switzerland.

^3^Department of Physics, P.O box 64, 00014 University of Helsinki, Finland.

^4^Laboratory of Atmospheric Chemistry, Paul Scherrer Institute, 5232 Villigen Switzerland.

^5^Institute for Atmospheric and Climate Science, ETH Zurich, 8092 Zurich, Switzerland.

^6^University of Innsbruck, Institute for Ion Physics and Applied Physics, Technikerstraße 25, 6020 Innsbruck, Austria.

^7^Finnish Meteorological Institute, Kuopio Unit, PL 1627, 70211 Kuopio, Finland.

^8^Department of Applied physics, University of Eastern Finland, P.O. Box 1627, FIN-70211 Kuopio, Finland.

^9^Department of Atmospheric Sciences, University of Washington, Box 351640, Seattle, WA 98195, USA.

^10^National Center for Atmospheric Research, P.O. Box 3000, 80307, Boulder, CO USA.

^11^CENTRA-SIM, University of Lisbon and University of Beira Interior, 1749-016 Lisbon, Portugal.

^12^Leibniz Institute for Tropospheric Research Permoserstr. 15 04318 Leipzig Germany.

^13^Faculty of Physics, University of Vienna, Boltzmanngase 5, 1090 Vienna, Austria.

^14^Center for Atmospheric Particle Studies, Carnegie Mellon University, 500 Forbes Ave, Pittsburgh PA 15213, USA.

^15^Department of Environmental Science, University of Eastern Finland, P.O. Box 1627, FIN-70211 Kuopio, Finland.

^16^California Institute of Technology, Division of Chemistry and Chemical Engineering, 1200 E. California Blvd Pasadena, CA 91125, USA.

^17^School of Earth and Environment, University of Leeds, LS2 9JT, UK

^18^Aerodyne Research Inc., Billerica, Massachusetts, USA.

**Contents of this file**

Figures S1

Tables S1

**Introduction**

In the supplementary information there is included Figure1. and Table 1. In our study, the theoretical evaluation of sulfuric acid monomer concentration was achieved by combining two theoretical approaches resulting in evaluation of sulfuric acid monomer and its clusters in respect to time. The sulfuric acid monomer and cluster concentration is evaluated by the use of ASAD (A Self-contained Atmospheric chemistry coDe) model and a modified version of SAWNUC (Sulfuric Acid Water NUCleation).The schematic displayed in Figure1 reveals the included parameters, the applied steps and the considerations involved in the evaluation of the final monomer and cluster concentration. For the theoretical evaluation of the monomer concentration of sulfuric acid using the ASAD model for atmospheric chemistry, the sulfuric acid production rate was evaluated taking into account all possible reactions of chemical tracers that represent the sulfuric acid gas phase oxidation in a system of SO_2_, O_3_ and H_2_O vapor in the CLOUD chamber. The possible reactants and chemical reactions leading to sulfuric acid production are displayed in Table 1.


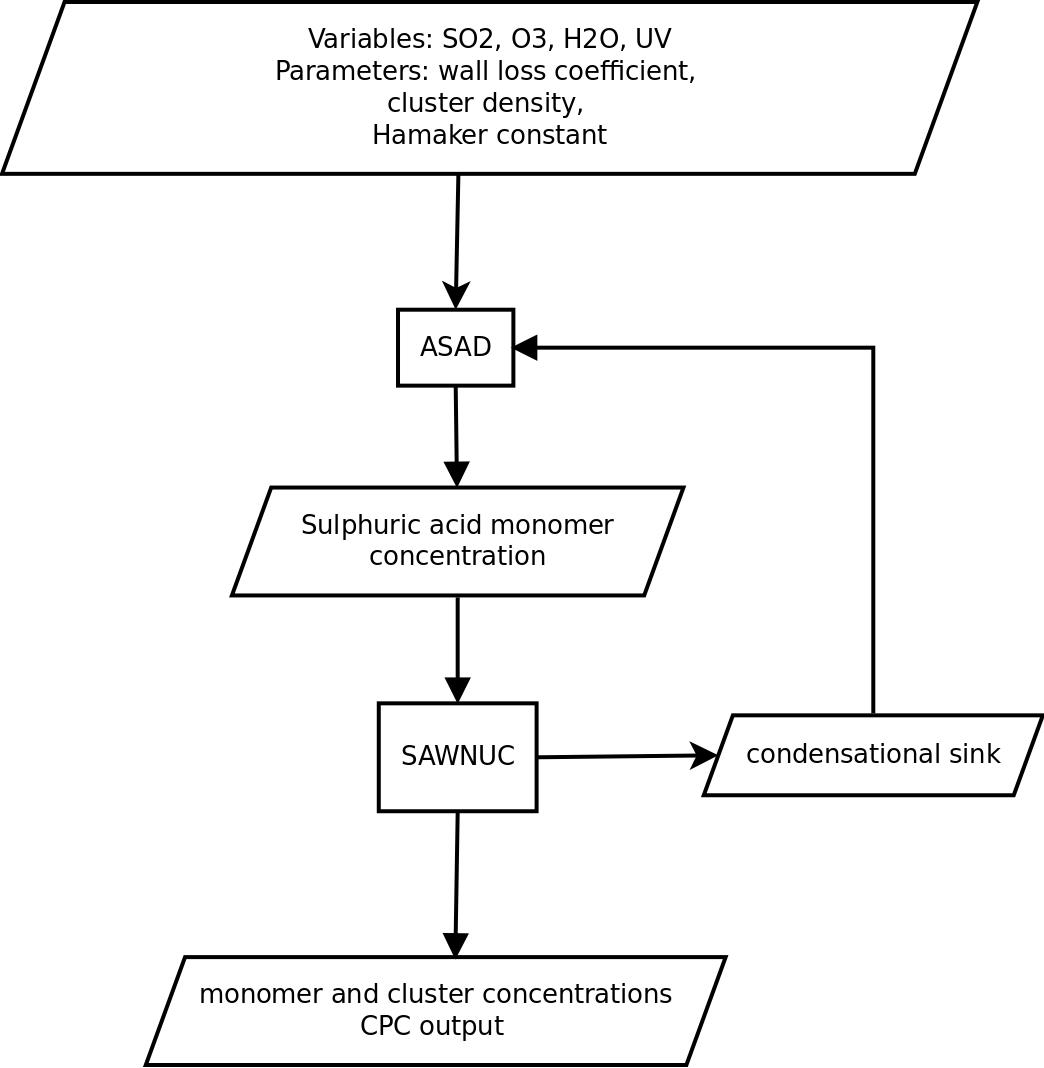


Figure S1. Schematic diagram for the sulfuric acid evaluation through the combination of ASAD and SAWNUC model.

| **Nr.** | **Reactant Reaction products** | |
| --- | --- | --- |
| 1  2  3  5  6  7  8  9  10  11  12  13  14  15  16  17  18  19  20  21  22  23  24  25  26  27  28  29  30  31  32  33  34  35  36 | H_2_O_2_ + PHOTON →  O_3_ + PHOTON →  O_3_+ PHOTON →  H + HO_2_ →  H + HO_2_ →  H + HO_2_ →  H + O_3_ →  HO_2_ + HO_2_ →  HO_2_ + O_3_ →  HOSO_2_ + O_2_ →  O(^3^P) + H_2_ →  O(^3^P) + H_2_O_2_ →  O(^3^P) + HO_2_ →  O(^3^P) + O_3_ →  O(^3^P) + OH →  O(^1^D) + H_2_ →  O(^1^D) + H_2_O →  O(^1^D) + N_2_ →  O(^1^D) + O_2_ →  O(^1^D) + O_3_ →  O(^1^D) + O_3_ →  OH + H_2_ →  OH + H_2_O_2_ →  OH + HO_2_ →  OH + O_3_ →  OH + OH →  SO_2_ + HO_2_ →  SO_2_ + O3 →  SO_3_ + 2H_2_O →  H + O_2_ →  HO_2_ + HO_2_ →  O(^3^P) + O_2_ →  O(^3^P) + SO_2_ →  OH + OH →  OH + SO_2_ → | OH + OH  O_2_ + O(^3^P)  O_2_ + O(^1^D)  H_2_ + O_2_  OH + OH  H_2_O+ O(^3^P)  OH + O_2_  H_2_O_2_ + O_2_  OH + O_2_ + O_2_  HO_2_ + SO_3_  OH + H  OH + HO_2_  OH + O_2_  O_2_ + O_2_  O_2_ + H  OH + H  OH + OH  O(^3^P) + N_2_  O(^3^P) + O_2_  O_2_ + O(^3^P) + O(^3^P)  O_2_ + O_2_  H_2_O + H  H_2_O + HO_2_  H_2_O + O_2_  HO_2_ + O_2_  H_2_O + O(^3^P)  OH + SO_3_  SO3 + O2  H_2_SO_4_ + H_2_O  HO_2_ + m  H_2_O_2_ + O_2_  O_3_ + m  SO_3_ + m  H_2_O_2_ + m  HOSO_2_ + m |

Table S1. List of reactions included in ASAD model.
